# Post-Operative Outcomes of Groin Hernia Repairs in Women: A Contemporary Review

**DOI:** 10.3390/diagnostics16070973

**Published:** 2026-03-25

**Authors:** Neha Soogoor, Setu Shiroya, Nana Kwadwo Okraku-Yirenkyi, Owen Falkenberg, Sonia Tripathy, Angel Sheu, Prem Kurra, Dhiresh R. Jeyarajah, Mohanakrishnan Sathyamoorthy

**Affiliations:** 1 Burnett School of Medicine at TCU, Fort Worth, TX 76104, USA; setu.shiroya@tcu.edu (S.S.); n.k.okraku@tcu.edu (N.K.O.-Y.); owen.falkenberg@tcu.edu (O.F.); sonia.tripathy@tcu.edu (S.T.); angel.sheu@tcu.edu (A.S.); 2 Apollo Institute of Medical Sciences and Research, Hyderabad 500096, Telangana, India; premk806@gmail.com; 3 Department of Surgery, Burnett School of Medicine at TCU, Fort Worth, TX 76104, USA; rohan.jeyarajah@tcu.edu; 4 Department of Medicine, Burnett School of Medicine at TCU, Fort Worth, TX 76104, USA; m.sathyamoorthy@tcu.edu

**Keywords:** postoperative, hernia, female, incidence, femoral, outcomes, diagnosis, interventions, repair

## Abstract

Abdominal wall hernias are common among men and women, often requiring surgical techniques to be managed safely and effectively. Abdominal wall hernias are typically classified as primary or secondary. Secondary hernias are most often related to prior surgical incisions. Primary hernias develop on their own and are not related to previous operations. Common primary hernias include inguinal, femoral, umbilical, and epigastric hernias, among others, with inguinal hernias being the most common. A narrative review was conducted to synthesize the current literature describing the incidence, epidemiology, surgical techniques, and post-operative outcomes of groin hernias in women using PubMed and Embase databases. Studies have demonstrated that women have an increased rate of recurrence of groin hernias, higher post-operative chronic pain, specifically when undergoing open repair. The discrepancies in diagnostic tools, outcomes, and treatment further highlight the need for studies surrounding women’s health and research in relation to surgical interventions to help inform clinicians’ management of groin hernias in the female population.

## 1. Introduction

**Figure 1:**
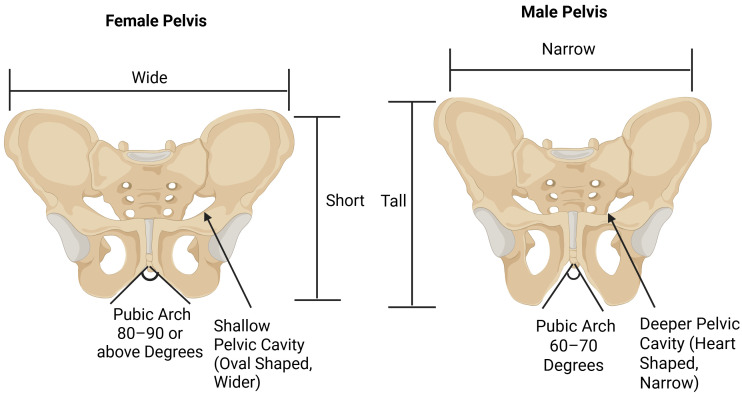
Created in BioRender. Shiroya, S. (2026) https://BioRender.com/3g5bul1. Comparison of female pelvic anatomy and male pelvic anatomy.

Hernias can be managed surgically, but the outcomes are often less favorable in women than men, as seen in both individual studies and large patient registries [1,2]. While making these important claims, it is important to recognize that the statements are based on limited female-specific data, as is seen in the paper written by Iyer et al. [3]. The most common types of hernias, ranked according to incidence, are inguinal, umbilical, epigastric, and femoral [4]. Inguinal hernias typically occur above the inguinal ligament and through the internal inguinal ring (indirect) or through Hesselbach’s triangle (direct). Femoral hernias occur through the femoral canal under the inguinal ligament and medial to the femoral blood vessels. Due to anatomical differences, women can often present with distinct types of groin hernias, especially femoral hernias. Women have a wider pelvis, which creates a larger femoral ring where a potential weak spot could occur [5]. The pelvic female and male anatomy dimensions are illustrated in Figure 1.

Hernias may entrap bowel loops and lead to incarceration, which results in progression to bowel obstruction or strangulation. When this happens, it is considered a surgical emergency. This is more common with femoral hernias due to the smaller opening in the abdominal wall [6]. The borders are also inflexible, with Cooper’s ligament inferiorly, lacunar ligament medially, and medial inguinal ligament superiorly. Due to these anatomical differences, physical examination in women can be more difficult. Thus, the round ligament serves as a critical anatomical landmark during both diagnosis and surgical repair. In general, women account for a small population of patients who undergo surgery for groin hernias, ranging from 8 to 11.5%. However, according to a retrospective analysis involving 181 females, the rate of emergency procedures in women is 3 to 4 times higher than in men, and this disparity is even more significant for femoral hernias, where the rate is 8 to 9 times higher [7].

Despite these significant risks, hernias are often underdiagnosed in the female population. This is further complicated by the clinical presentation and treatment of hernias, which include open and minimally invasive surgery, which can result in discrepancies in post-operative outcomes between men and women.

Currently, the literature addressing these groin hernias in males is abundant; however, the breadth of studies surrounding diagnosis, treatment, and post-operative outcomes in women is limited. This study aims to address the gaps in the literature and highlight the current data available to guide clinical reasoning and future research.

## 2. Methods

This study was conducted as a narrative review examining the sex-based differences regarding groin hernias, post-operative outcomes, and repairs. A focused literature review was conducted using PubMed and Embase databases, with key search terms including, but not limited to, “groin hernias”, “femoral hernia”, “female-sex”, “women”, “inguinal hernia”, “laparoscopic technique”, “hernia repair”, “chronic pain”, “recurrence”, and “reoperation”. Multiple Boolean combinations of keywords were used in the various databases per the appropriate syntax. The literature from 1997 to 2025 was considered to adequately capture both historic and current findings on the topic. Studies were selected to include level 1, level 2, level 3, and level 4 evidence to evaluate past and current literature available. This study involved analysis and synthesis of literature on anatomy, epidemiology, groin hernia repair methods, with exclusion of robotic surgeries, as it was not within the scope of the review, and post-operative outcomes. This study did not include an institutional board review. Data were extracted from included studies, and statistically significant results were synthesized and presented to emphasize sex-related differences in groin hernia repairs. GenAI was not used in the generation of text, data, graphics, or study design, or for data collection, analysis, or the interpretation of data. GenAI was only used for the purposes of editing original text (grammar, spelling, and punctuation).

## 3. Epidemiology of Hernias

According to the HerniaSurge Group, a global group of expert surgeons with experience in hernia surgery and research, groin hernias are 8–10 times more common in men than women [8]. Though they are not uncommon in women, with a lifetime risk of 3–6%. However, many studies of hernias in women are often limited to hernias in pregnancy or rare hernias. Although inguinal hernias are more common in men, women are at higher risk of having recurrent inguinal hernias and femoral hernias [9]. This risk can be attributed to the greater distance between the pubic tubercle and the internal ring and the wider rectus abdominis muscle in females [8]. Since inguinal hernias may be more difficult to assess via physical exam in women, CT scans are a helpful modality with a sensitivity of 100% for femoral hernias [10]. Furthermore, inguinal hernias can also only involve the herniated preperitoneal fat known as the canal lipoma, which can be mistaken for a hernia. Due to a lack of a peritoneal hernia sac in these women, these hernias must be dissected in the operating room to be confirmed as a diagnosis.

Laparoscopic surgery allows for direct visualization of all hernia types, and, in high-income countries, laparoscopic repair demonstrates superior outcomes, including a decrease in reoperation rates in women [10]. Danish nationwide data demonstrated that laparoscopic repair reduced reoperation risk by 67% compared to open repair (HR 0.33, 95% CI 0.09–0.95) [11]. However, in low-income countries such as Uganda, laparoscopic approaches are limited due to cost, and groin hernia repair in women is predominantly performed using suture techniques rather than mesh repairs [12]. There is a major literature gap when comparing the low-income and high-income diagnostic modalities, which is an important topic of future research due to limitations in resources.

Furthermore, current evidence suggests that a family history of inguinal hernias has been demonstrated to be an associated risk factor in women. One case–control study identified several factors associated with inguinal hernias in women. Where women with a family history of inguinal hernias were over four times more likely to develop one (OR = 4.3, 95% CI 1.9–9.7) [13]. Furthermore, a systematic review performed by Oberg et al., including 22 studies with unique participants, showed that children were more likely to develop an inguinal hernia if their parents or siblings had an inguinal hernia, with the strongest link seen between mothers and daughters and between sisters. In adults, those with an inguinal hernia were over five times more likely to have a positive family history for the condition compared to those without a hernia [14]. Women with chronic constipation had a 2.5 times higher risk of developing an inguinal hernia due to increased intra-abdominal pressure (OR = 2.5, 95% CI 1.0–6.7) [13]. The method of primary repair can also increase the rate of recurrence and reoperation in cases of femoral hernia. Studies from the Danish Hernia Database and the Swedish Hernia Registry all concluded that recurrence rates were lower with mesh repair as compared to tissue repair. Studies also show that preperitoneal mesh repair yields better results than plug repair. Currently, all repairs use mesh as common practice [8].

Protective factors against inguinal hernias in women include high levels of current sports activity and obesity. Active engagement in sports lowers the risk of inguinal hernias in women (OR = 0.2, 95% CI 0.1–0.7), likely due to increased strength of the abdominal wall muscles [10]. Surprisingly, a BMI > 30 can also be protective since there is more support to the abdominal wall [13]. Preperitoneal fat in obese individuals fills and supports potential hernia defects. This ‘padding’ effect makes regular hernia signs less apparent and increases the possibility of obese patients presenting with incarceration and strangulation. However, obese patients also face the challenge of difficult examination and diagnostics for hernias, which counteract the protective factors that preperitoneal fat can provide [8].

## 4. Groin Hernia Repair Methods

Groin hernia repair methods include open and minimally invasive surgery, with minimally invasive surgery. The postoperative outcomes for these repairs vary and should be taken into consideration when selecting an operative approach for individual patients.

There are multiple methods for open repair of inguinal hernias. Surgical options utilizing mesh include Lichtenstein repair, plug and patch, preperitoneal, and a combined approach (onlay + underlay). The Lichtenstein repair is considered “tension-free” and involves the placement of a flat mesh prosthesis over Hesselbach’s triangle. The mesh is secured with interrupted sutures to the inguinal ligament, conjoint tendon, and pubic tubercle. The mesh has a slit to accommodate the spermatic cord [15]. The Lichtenstein repair avoids suture line tension and has become the gold standard for open inguinal hernia repair. In the Lichtenstein repair, the round ligament is preserved and is essential to reduce the risk of injury to the genital branch of the GNF Nerve [16]. Mesh coverage can be difficult around the round ligament. Improper mesh coverage can lead to high risks of medial recurrence near the pubic tubercle. To address femoral hernias, novel surgical techniques have been introduced for Lichtenstein repairs. The extended Lichtenstein repair utilizes a self-gripping mesh and is folded over the Cooper’s ligament. It will cover the femoral orifice and extend laterally over the femoral vessels. In a study comparing an extended Lichtenstein repair for femoral hernias and an original Lichtenstein repair for non-femoral inguinal hernias, no wound-related complications and no recurrence were observed after 32 months in the femoral hernia group. Out of 50 femoral hernia patients, 12 were female. Their findings suggest that intraoperative digital palpation is a reliable method for identifying femoral hernias [17]. The femoral canal is not routinely explored in the Lichtenstein procedure, so a “false” recurrence with a missed femoral hernia is possible. This method is also associated with mesh migration and erosion and is not recommended. Given the anatomical predisposition of female patients to femoral hernias, routine exploration of the femoral canal should be a mandatory component of the Lichtenstein procedure in women. This is critical to mitigate the significant risk of a missed primary femoral hernia and reduce the disproportionately high recurrence rate observed in women.

The preperitoneal open inguinal hernia repair involves placing the mesh in the preperitoneal space, deep to the transversalis fascia and anterior to the peritoneum to reinforce the myopectineal orifice. The open procedure can be done via various approaches, including a transinguinal method such as the Rives operation, a slit made in the abdominal muscles (Wantz and Kugel repairs), or a lower midline abdominal incision (the Stoppa GPRVS method). Complications include injury to pelvic structures such as major blood vessels, bowel, and bladder [18,19].

The combined approach (onlay plus underlay) utilizes two layers of mesh placement. The Prolene Hernia System utilizes a combined approach. The onlay mesh is placed over the inguinal floor and reduces the likelihood of recurrence. The underlay mesh is placed behind the abdominal wall muscles in the preperitoneal space. The two layers are connected by a mesh bridge, a connector that secures the underlay patch and reduces the incidence of migration [20]. Complications are comparable to those of other mesh techniques.

These repairs are not suitable for femoral hernia repairs. The Lichtenstein repair places mesh over the inguinal floor and does not cover the femoral canal. There is a high risk of missing a femoral hernia and increased recurrence rates in women [8]. The open preperitoneal and combined approaches present with anatomical challenges and coverage difficulties in women. Femoral hernias present as a small size and deep location compared to inguinal hernias, making them easy to miss during an open procedure. However, femoral hernias are larger in women compared to men, due to wider pelvis size and can lead to a higher risk of strangulation. For reference, the adult male pelvis is narrower, exhibiting an oval or heart-shaped pelvic inlet. The angle of the pubic arch is less than 90 degrees. The adult female pelvis is broader and exhibits a round pelvic inlet. The angle of the pubic arch is greater than 90 degrees. Another study has used a modified version of the Lichtenstein repair with the Prolene Hernia System, which is anatomically targeted toward the inguinal region and does not provide adequate coverage of the femoral canal. Preperitoneal mesh repairs can be adapted to femoral hernias, but again, are not specifically designed for them without modification [21]. In these cases, the mesh is attached to Cooper’s ligament and covers more surface area to reduce the recurrence of femoral hernias.

The Shouldice repair is an inguinal hernia repair that uses no mesh, unlike the Lichtenstein repair. Instead, it utilizes the patient’s own tissue, considered a “tissue-repair”. After the hernia sac is excised or reduced, the transversalis fascia is opened. A four-layer reconstruction utilizing two continuous sutures is performed. The transversalis fascia, internal oblique, and external oblique aponeurosis are sewn together in a laminated fashion [22]. The Shouldice repair preserves the round ligament by retracting it to tighten the internal ring and inguinal floor. Compared to the Lichtenstein repair, a randomized trial showed that there was a significant difference in operating time in favor of the Lichtenstein technique [23]. Another randomized trial favored Lichtenstein repair in lower complication and recurrence rates. In 331 procedures, there was a 2% incidence of recurrence for the Shouldice repair. In 371 procedures, there was a 0.5% incidence of recurrence for Lichtenstein [24]. However, the Shouldice repair technique is highly specialized and requires specially trained surgeons to master. Therefore, it is highly irreproducible in practice [25,26]. Lichtenstein repair remains a widely practiced technique in men due to the decreased rate of recurrences; however, women have been studied to have high rates of recurrence as compared to laparoscopic repair, with 40.9% of the repairs being due to missed femoral hernias in open repair [27]. Another non-mesh-based repair includes the McVay/Cooper’s ligament repair, which can be specifically used for femoral hernias. Sutures are placed that approximate the transversus abdominis arch to Cooper’s ligament, starting at the pubic tubercle and moving laterally. At the medial edge of the femoral ring, transition sutures are used to incorporate the iliopubic tract. This closes the femoral canal and reinforces the floor of the inguinal canal. However, they result in high tension. The Cooper ligament and inguinal ligament lack elasticity, and sutures may break, resulting in recurrence [28]. However, one study compared mesh repair and suture repair for the treatment of incarcerated femoral hernia; 36 out of 48 patients were female, and 27 of them underwent a McVay repair. The study found that there was no significant difference (*p* > 0.005) in the recurrence rates, postoperative infection, chronic pain, or operation duration between the suture and mesh repairs for femoral hernias [29].

A large consensus on the frequency of open inguinal hernia repair techniques is unknown; however, a smaller consensus can give clues as to how often each technique is used. In 2019, the Americas Hernia Society Quality Collaborative, out of 4613 patients, 42% were repaired using an open technique. Of the open repairs, 8% were tissue-based repairs; the Shouldice repair was the most common tissue-based repair. The other 92% were mesh-based repairs; the Lichtenstein repair was the most common mesh-based repair (57%) [30]. Overall, a variety of open inguinal hernia repair techniques are available, each with its own advantages, limitations, and considerations for patient selection.

### Minimally Invasive Approach to Groin Hernia Repair

When considering surgical treatment options for inguinal hernias, the utilization of mesh has been strongly recommended. Within mesh repair, there are two approaches: an anterior and a posterior approach [8]. According to the European Hernia Society, all male adults > 30 years who have a symptomatic hernia should be operated on with mesh, with the best approach endoscopically being the posterior approach. These approaches are often the transabdominal preperitoneal (TAPP) approach and the totally extraperitoneal (TEP) approach. The TAPP approach includes entering the abdominal cavity to place a mesh anterior to the peritoneal lining [31]. During a totally extraperitoneal (TEP) approach, the mesh is also placed in front of the peritoneal lining; however, the peritoneal cavity is not entered during this procedure. Since 1990, the posterior endoscopic approach has been conducted via the Stoppa technique for both TAPP and TEP approaches. A TEP approach requires dissection using a balloon in the preperitoneal space, which can be technically challenging, and a TAPP approach can result in adhesions in the abdomen. Laparoscopic surgery for groin hernia repairs has allowed for a minimally invasive approach, resulting in reduced complication rates, low chronic pain risk, and is often cost-effective [8]. Debate exists about which approach is more effective and minimizes the risk of complications [31]. Typically, endoscopic repair has been documented to be the superior approach when considering chronic pain, as well as recommended for bilateral inguinal hernia repairs. It is also cost-effective for the working population [32].

In females, it is recommended that a preperitoneal (endoscopic) approach be used for the treatment of female hernia due to the perceived ease of covering the femoral and inguinal orifices simultaneously using mesh [8,32]. Currently, the standard of care includes mesh unless there is a considerable contraindication. However, covering the orifices simultaneously can be difficult due to the inability to discern distinctions between inguinal and femoral hernias, especially in obese women, resulting in femoral hernias becoming overlooked during open anterior repair. Due to this higher risk of femoral hernias, the TAPP approach can be highly useful in groin hernia repair in women. It is also important that timely repair of groin hernia occurs, as mortality risk and strangulation risk increase greatly with increased watchful waiting in women [8,32]. Anatomical differences should also be considered when choosing surgery of choice. There has been debate surrounding the approach to the round ligament and whether it should be divided or not. Evidence surrounding the debate is highly limited; however, in laparoscopic surgery, division is optional but does provide for better mesh placement, with fewer complications in the preperitoneal space. In comparison, the round ligament is not typically divided in open repair as it can result in division of the genital and ilioinguinal nerve, resulting in complications such as deafferentation hypersensitivity and ipsilateral labial numbness [8].

**Figure 2:**
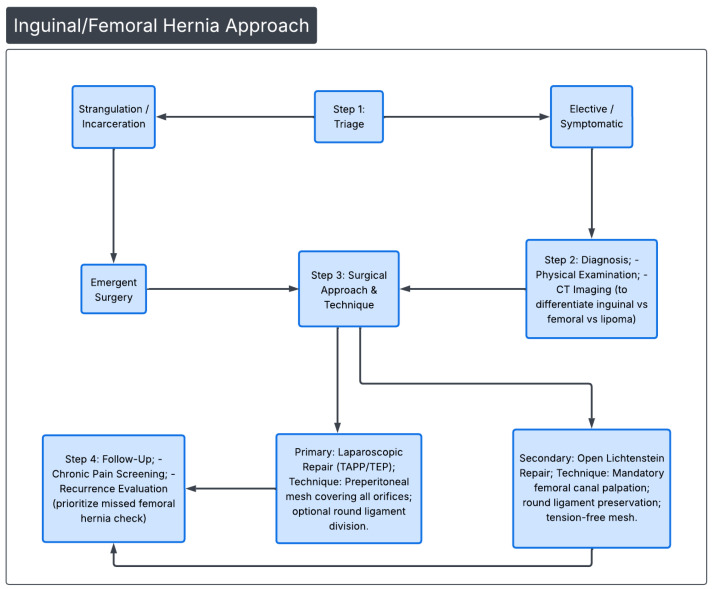
Referenced from Section 4 and Section 5 (Groin Hernia Repair Methods and Post-Operative Outcomes). Condensed clinical approach to groin hernias, suggested repairs, and follow-up prioritizing checking for femoral hernias.

For women undergoing elective or emergency hernia repair, it is recommended that the laparoscopic approach be used, as it is more likely to identify a concurrent femoral hernia as well. Females are at high risk of developing a femoral hernia, with incarceration and strangulation being common complications [33]. In a prospective study by Koch et al., involving over 6500 women, 46% of women were found to have a concurrent femoral or obturator hernia while undergoing repair of a recurrent inguinal hernia [33]. While this data is older, we believe it to be important in recognizing the chronicity of this issue. However, a 2019 study including over 200,000 patients with inguinal hernias demonstrated that women were less likely to undergo a laparoscopic repair at 31.58% compared to males at 41.43%, further suggesting that diagnosis may need to be studied in females to improve their clinical outcomes [34]. These types of hernias are four times as common in women over 50 years old, often requiring emergent or elective repair. The laparoscopic approach is the preferred option for surgical repair of groin hernias in women and is highly recommended, provided that the surgeon has expertise in performing the technique [8,32]. A condensed algorithm illustrating the diagnostic approach and surgical techniques is demonstrated in Figure 2.

## 5. Post-Operative Outcomes

Surgical repair of groin hernias encompassing inguinal and femoral subtypes carries important postoperative considerations, including recurrence, chronic pain, and secondary symptoms. Although inguinal hernias are more prevalent in men, women with groin hernias experience disproportionately higher morbidity [8,32]. This includes increased rates of recurrence, greater incidence of missed femoral hernias at the time of repair, and worse long-term pain outcomes. These disparities are compounded by the historical underrepresentation of women in surgical hernia trials, which has led to a lack of sex-specific outcomes data [32].

### 5.1. Recurrence

#### 5.1.1. Comparison by Sex

While men account for the majority of groin hernia cases, recurrence after repair is significantly more common in women. In a Swedish cohort evaluating 6895 female groin hernia repairs, early reoperations were frequently attributable to femoral hernias that had not been identified during the index surgery [33]. Femoral hernias are more prevalent in women, and their underdiagnosis contributes to apparent inguinal hernia “recurrences” when, in fact, a separate femoral defect was likely missed (*p*-value = 0.0006). Data from the European Hernia Society confirm that a proportion of nearly 40% of reoperations in women after groin hernia repair are due to missed femoral hernias in initial groin hernia repair [1]. Furthermore, registry studies report that women have a higher reoperation rate than men following both mesh and non-mesh open repairs, regardless of hernia type [8].

#### 5.1.2. Comparison by Technique in Women: Open vs. Laparoscopic

Among women, laparoscopic approaches have consistently demonstrated lower recurrence rates compared with open anterior repairs. A systematic review reported a recurrence rate of 1.2% after laparoscopic repair versus 4.9% after open repair, with 41% of recurrences after open repair attributable to missed femoral hernias [27]. National data similarly show significantly lower reoperation rates in women who underwent laparoscopic femoral hernia repair compared with open repair [11]. Contemporary international guidelines strongly recommend a laparoscopic approach in all female patients undergoing groin hernia repair to reduce recurrence and improve anatomic accuracy [8].

#### 5.1.3. Round Ligament Management in Female Groin Hernia Repair

The round ligament of the uterus is a female-specific structure traversing the inguinal canal and encountered during groin hernia repair. Its management may influence both recurrence and postoperative pain outcomes [8,32]. During laparoscopic repair, the ligament is often divided to aid mesh placement; however, no significant differences in chronic pain, sensory disturbances, or recurrence rates have been found between division and preservation [35]. Guidelines recommend preserving the round ligament where feasible, particularly during laparoscopic repair [8]. Strategies to preserve the ligament include lateralization or passage through a mesh slit to maintain anatomical continuity without disrupting prosthetic positioning. In contrast, open repairs typically do not require ligament division, as the anterior approach allows for retraction or dissection around the structure without sacrificing it [32]. Despite these considerations, round ligament management remains inconsistently reported in surgical studies and often omitted from operative notes. If any doubt remains, it is typically divided to cover the orifices.

One strategy that has been studied is the decision to transect the round ligament intraoperatively, which has yielded varying results. A study of 1365 female patients performed by Renshaw et al. examined postoperative results among patients who received round ligament transection vs. not and found no difference in quality-of-life scores 30 days post op, but a lower mean pain score at 6 months in patients who received a round ligament transection [36]. However, a meta-analysis comprising 1131 women found no difference in postoperative pain or paresthesia with a transection of the round ligament compared to not [36]. These conflicting results continue to emphasize the importance of gender specific and individualized treatment plans and further investigation of this technique for greater success rates.

### 5.2. Chronic Pain Following Groin Hernia Repair

Chronic postoperative pain, defined as pain persisting for more than three months after hernia repair, is a well-recognized complication that significantly affects long-term quality of life. Given the overlap in clinical presentation and contributing factors, chronic pain and related postoperative sensory or activity-linked symptoms are discussed together in this section to reflect their shared relevance during follow-up. Both sex and surgical technique independently influence the risk and severity of chronic pain, with women and patients undergoing open repair experiencing disproportionately worse outcomes.

Although chronic pain is often operationalized using a time threshold, postoperative groin pain after hernia repair is heterogeneous in both phenotype and clinical impact. International guidance recommends retaining a three-month postoperative threshold while also incorporating patient-centered severity, specifically pain that is at least moderate and interferes with daily activities [8]. This framing matters because clinically significant chronic postoperative groin pain is not rare, tends to decrease over time, and is influenced by both patient-level factors and operative approach [8]. In particular, female sex and open repair are recognized risk factors, which underscores why pain outcomes in women require deliberate attention when selecting technique and interpreting postoperative symptoms during follow-up [8].

Mechanistically, chronic postoperative inguinal pain is often conceptualized as a combined sequence of mesh-tissue biology and local neuroanatomic vulnerability. Mesh implantation triggers a foreign-body reaction with inflammatory and fibrotic cellular responses, and persistent inflammation can promote ongoing collagen deposition and scar accumulation, creating a substrate for tethering and irritative symptoms in the groin [8]. Within that environment, guideline discussion emphasizes preventable technical contributors that can amplify neuropathic pain patterns, including suture entrapment of nerves and mesh-stimulated scarring, which helps explain why similar repairs can yield different long-term pain phenotypes across patients. This etiologic framework provides context for the sex- and technique-stratified outcome comparisons that follow.

In addition to chronic pain, many patients—particularly women—experience secondary postoperative symptoms that do not meet criteria for chronic neuralgia but still impair quality of life. These include intermittent groin discomfort, positional tenderness, exercise-induced pain, cutaneous allodynia, paresthesia, and, in some cases, referred pelvic or thigh pain. Such symptoms are often underreported in the literature and underrecognized in clinical follow-up, despite their significant functional impact.

#### 5.2.1. Comparison by Sex

Numerous studies have demonstrated that women experience higher rates of chronic pain after groin hernia repair compared with men. A systematic review and meta-analysis were conducted that showed women had higher rates of post-operative pain following inguinal hernia repair. Women experienced a 20.4% increase compared to men at 10.4%, with female sex being independently associated with increased risk (OR 1.89, 95% CI 1.02–3.47; *p* < 0.05) [37]. These differences have been attributed to sex-specific anatomical and physiological factors, including more diffuse nerve distribution in the inguinal region, smaller nerve diameters, and greater central pain sensitivity [38]. In both cohort studies and large patient registries, female sex has historically and consistently emerged as an independent predictor of chronic postoperative pain after groin hernia repair [39,40].

Secondary pain syndromes, including intermittent groin discomfort, positional tenderness, exercise-induced pain, cutaneous allodynia, paresthesia, and referred pelvic or thigh pain, are more frequently reported by women than men following groin hernia repair. These symptoms often do not meet criteria for chronic neuralgia but still significantly impair quality of life and are often underrecognized in clinical follow-up [8,41]. Factors contributing to this higher prevalence in women include the presence of the round ligament, greater variability in pelvic fascial anatomy, and heightened baseline central pain sensitivity [38]. Women have also been shown to experience higher rates of femoral hernias following groin hernia repair [8]. These patterns suggest that secondary pain is not only more common in women but also more varied in presentation.

#### 5.2.2. Comparison by Technique in Women

Among women, surgical technique significantly influences the likelihood of developing chronic pain. A systematic review and meta-analysis published in the American Journal of Surgery showed that open anterior mesh repairs, particularly the Lichtenstein technique, are associated with higher rates of postoperative neuralgia, most commonly attributed to nerve irritation in the setting of mesh fixation and scarring, including entrapment and perineural fibrosis involving the ilioinguinal, iliohypogastric, or genitofemoral nerve territories, with direct nerve injury and tissue inflammation as additional potential contributors [42,43,44]. In contrast, laparoscopic approaches such as transabdominal preperitoneal (TAPP) and totally extraperitoneal (TEP) repair have been associated with significantly lower chronic pain rates. In a five-year randomized controlled trial, male patients who underwent laparoscopic repair reported fewer pain symptoms and reduced analgesic use compared to those who received Lichtenstein repair [45]. The minimally invasive approach allows for mesh placement in the preperitoneal space and reduces direct manipulation of sensory nerves, thereby minimizing the risk of entrapment and fibrosis-induced neuropathy. Because inguinodynia is commonly attributed to ilioinguinal nerve irritation, elective division of the ilioinguinal nerve has been proposed and studied as a preventive strategy to reduce chronic groin pain. However, a meta-analysis of nine randomized controlled trials did not show a reduction in groin pain incidence with routine division compared with preservation [46]. The evidence is predominantly from male cohorts, and needs to be further studied in women, particularly given sex-specific anatomic considerations and the broader heterogeneity of postoperative pain phenotypes reported in women. Given this evidence, laparoscopic repair is generally preferred in women with groin hernias to reduce the burden of chronic postoperative pain. Proper preoperative counseling and tailored surgical planning should reflect both sex-specific risks and the protective effects of technique selection.

Surgical technique influences the incidence and character of secondary pain outcomes. Open anterior mesh repairs, particularly when using sutures for mesh fixation, are associated with increased rates of localized tenderness and positional discomfort due to superficial nerve irritation and scar tethering [47]. Laparoscopic techniques, by contrast, involve deeper mesh placement and generally result in fewer cutaneous sensory disturbances. In comparative studies, patients undergoing laparoscopic transabdominal preperitoneal repair reported lower rates of groin discomfort during physical activity than those who had open repairs [48]. The reduced manipulation of superficial nerves and better visualization of neurovascular structures likely contribute to this benefit. Additionally, laparoscopic repair provides more consistent coverage of femoral and obturator spaces, reducing the risk of referred pain from missed defects or improperly positioned mesh [32]. Given the heterogeneity of secondary pain syndromes and the difficulty in capturing them with conventional pain scores, clinicians should maintain a high index of suspicion for these outcomes in postoperative women, particularly when presenting with vague or dynamic pain patterns.

### 5.3. Reoperation and Surgical Site Complications Following Groin Hernia Repair

Reoperation is a clinically significant postoperative outcome that reflects both technical failure and complications such as recurrence, infection, or mesh-related morbidity. Emerging evidence highlights sex-based differences in reoperation rates, with women generally experiencing higher reoperation risk than men following groin hernia repair. These disparities are influenced by both the type of hernia and the surgical technique used.

#### 5.3.1. Comparison by Sex

Women have higher cumulative reoperation rates than men after groin hernia repair, particularly following open anterior approaches. Data show reoperation rates of 4.0% in women compared with 2.1% in men after open repair, while laparoscopic repairs in women are associated with a lower reoperation rate of 1.9% compared to 2.6% in men [8,11]. The increased reoperation risk in women is attributed to the higher prevalence of missed femoral hernias during the initial procedure [33].

#### 5.3.2. Comparison by Technique

Laparoscopic repair is also associated with lower rates of superficial wound complications, such as seroma, hematoma, and surgical site infection, compared to open repairs [8]. Open approaches, by contrast, are more frequently associated with early postoperative complications, including hematoma formation in up to 22% of patients and a higher incidence of surgical site infections [11]. Taken together, these data reinforce the importance of sex-specific and technique-sensitive planning in groin hernia surgery. In women, laparoscopic repair offers both a diagnostic and therapeutic advantage by reducing reoperation rates and postoperative complications.

### 5.4. Mesh-Related Complications Following Groin Hernia Repair

Mesh-related complications, including local discomfort, erosion, and infection, are important considerations in groin hernia repair, particularly in women due to sex-specific anatomical factors. Women report higher rates of foreign body sensation or non-specific groin discomfort after mesh-based repairs [48]. Plug mesh techniques, which require deeper placement into anatomical spaces, are associated with an increased risk of erosion and migration, especially in women. These techniques have largely fallen out of favor [8]. Open mesh repairs are linked to localized discomfort and potential nerve tethering, while laparoscopic techniques allow for preperitoneal placement with fewer fixation points, reducing nerve contact and improving conformity of the mesh to anatomical planes [48]. Lightweight, large-pore meshes have been shown to reduce stiffness and foreign body sensation compared to standard meshes [49]. Post-operative outcomes for this section have been outlined in Table 1.

### 5.5. Pathobiology and Genetic Risk Factors

Extensive research pertaining to clinical and demographic factors that influence surgery outcomes between men and women exists. However, currently, there is minimal investigation into genetic factors that could be placing women at higher risk and providing potential data to inform physicians when selecting treatment modalities. With women at a higher risk due to family history, it is reasonable to theorize that there are potential pathobiological factors contributing to the surgery outcomes women experience. A systematic review published in the American Journal of Surgery found an increase in matrix metalloproteinase-2 (MMP-2) in patients with detectable inguinal hernias [50]. Additionally, compared to hernia-free controls, this study found that type 1 collagen synthesis is reduced, type 1/type3 collagen synthesis ratio is reduced, and markers of type IV collagen synthesis are increased [50]. Finally, other molecular markers such as IGF-1, IGFBP and cortisol were shown to be greater in patients with inguinal hernias [50]. The intriguing results offer an opportunity for prospective studies to be implemented, while adding to the compounding information of hernia pathology, but do not offer pre-hernia levels of the markers. A prospective study stratified both by sex and molecular markers, following patients who both develop inguinal hernias and do not, could be beneficial in creating preoperative and postoperative treatment plans. Furthermore, obtaining family data on patients as well, stratified by the history of inguinal hernia, would help correlate and stratify the true impact of family history, i.e., a unigenetic or polygenetic basis impacting clinical outcomes for women with inguinal hernias [51]. It is intriguing to imagine whether pathogenic alterations in collagen-encoding genes are associated with an increased risk of developing inguinal hernias.

## 6. Discussion

Understanding the risk factors involved in groin hernias, their repair, and their recurrence rates is important to optimize treatment and monitoring strategies for patients, specifically women at high risk. Family history has been identified as a risk factor for groin hernias in women, and with the available data described, it is shown that women have a higher rate of groin hernias, higher reoperation rates, and increased pain following surgery [13,40]. In particular, the rate of emergency groin hernia procedures highly implicates the clinical decision-making in women. Studies have shown a substantially higher rate of emergency groin hernia procedures than in men, approximately 14.5–17.0% compared to 3.0–5.1% in men. Furthermore, the rate rising to as high as 40.6% in the presence of a femoral hernia highlights the increased risk of incarceration and strangulation in female patients [52]. Additionally, femoral hernias are frequently diagnosed at the time of emergency presentation, exhibiting incarceration rates of 40–80% in some studies [53]. Expanding upon this knowledge can assist clinicians in developing personalized treatment plans and identifying patients at risk who would benefit from increased monitoring.

Further, using evidence to select the best surgical option is important in optimizing patient outcomes. Laparoscopic repairs have been shown to be more advantageous in women compared to men when considering reoperation rates and, thus, should influence surgical decision-making. However, this statement is underpowered due to a significant gap in female-specific studies that limits providers’ choices in selecting an optimal surgical approach. This gap has created guidelines based on male-dominated studies, which may be an inappropriate l choice and structure for repairs in women. Female-only studies like this, and others, must be completed to better serve this patient population. One study aimed to reduce this gap recommended the use of the TEP and TAPP techniques to repair hernias vs. the Lichtenstein repair because they proved to be superior [2]. Expansion of knowledge, such as this study, is encouraging, but still, there are few studies investigating in a female-specific capacity, leaving much work to be done in the pursuit of gender-focused guidelines. In the pursuit of improved patient satisfaction and safety, we propose further investigation into post-operative chronic pain in women. As mentioned prior, the division of the ilioinguinal nerve has not improved pain outcomes compared to preservation—a finding described from largely male-dominated studies [46]. We propose a prospective analysis of post-operative pain management in women when comparing ilioinguinal nerve division or preservation. Expanding the literature base in this way emphasizes the importance of sex specific outcomes and assists in building sex specific guidelines in the pursuit of optimal patient outcomes. Furthermore, a study by Rasador et al. reported that only 20.7% of women received minimally invasive approaches like laparoscopic repair for groin hernia repair, and 79.3% received open approaches despite guidelines recommending minimally invasive approaches [54]. We propose a survey that investigates barriers to receiving minimally invasive surgery in women. These could include many components, such as socioeconomic factors as well as hospital factors (i.e., community vs. private structure). We believe this investigation should be further extrapolated into lower- and middle-income countries (LMIC). If women are to be served to the full extent possible, it is crucial to involve the global totality of healthcare. While there are claims that minimally invasive surgery is more expensive, its association with significantly reduced reoperation rates suggests the proposed higher up-front cost is worth it when concerning long term complications and costs. By determining factors that may prohibit women from receiving optimal surgical treatment, we can provide knowledge to providers and implement safeguards to prevent post-operative complications from as early as the initial patient presentation. While we believe this review to be comprehensive, we do acknowledge limitations in our investigation. The use of registry data can be incomplete and is vulnerable to bias. The registries can have missing data on certain patients included, are privy to selection bias and often rely on clinician-reported outcomes rather than standardized assessments at pre-defined intervals. Additionally, retrospective studies and patient registries are vulnerable to confounders such as non-standardized pain reporting, age differences and surgeon experience. These factors must be considered when interpreting large-scale results in patient registries.

In summary, incorporating a holistic view of risk factors and primary repair techniques into practice will enhance management and care for women with and at high risk of inguinal hernias. Specifically, addressing the data gap with respect to hernias will greatly contribute to creating protocols that emphasize the safety, satisfaction, and recovery outcomes tailored to women’s unique needs.

## 7. Conclusions

The risk factors for women who experience hernia recurrence are slowly becoming more well-defined, but still highlight a clear gap in our knowledge about their incidence. On the contrary, it has been identified that both the TEP and TAPP techniques for repair have proved to be superior and beneficial in women in reducing recurrence rates [2]. While the review in this article identified various studies that describe factors detrimental and beneficial to women, the data continues to be incomplete compared to the male dataset counterpart. Therefore, the continued study of hernia recurrence in women is an issue that is solvable, relevant, and important in modern-day medicine. The current literature lacks prospective studies focused on optimizing outcomes for women to create guidelines tailored strictly for women. To create better care, it is crucial to begin these investigations to further specialized medicine for women.

## Figures and Tables

**Table 1:** Outcomes in open anterior repair vs. laparoscopic repair.

Outcome	Open Anterior Repair (e.g., Lichtenstein)	Laparoscopic Repair (TAPP/TEP)
Recurrence Rate	4.9% (41% of which are missed femoral hernias)	1.2%
Chronic Pain Incidence	Higher; associated with postoperative neuralgia, often linked to nerve entrapment and perineural fibrosis	Significantly lower; reduced analgesic use and nerve manipulation
Reoperation Rate	4.0%	1.9%
Wound Complications	Higher; hematoma in up to 22%, higher infection risk	Lower; fewer seromas, hematomas, and infections
Secondary Pain	Increased localized/positional tenderness and scar tethering	Fewer cutaneous sensory disturbances and groin discomfort

Referenced from Section 5. Exact pooled percentages for Chronic Pain Incidence, Wound Complications, and Secondary Pain in women specifically are unavailable in the current literature, serving as a limitation.

## Data Availability

No new data were created or analyzed in this study. Data sharing is not applicable to this article.

## Abbreviations

The following abbreviations are used in this manuscript:

TAPP	Transabdominal preperitoneal
TEP	Totally extraperitoneal
